# Does e-cigarette use affect response to non-surgical periodontal therapy?

**DOI:** 10.1038/s41432-023-00947-8

**Published:** 2023-10-23

**Authors:** Satish Kumar, Marc Shlossman

**Affiliations:** Arizona School of Dentistry & Oral Health, Mesa, AZ USA

**Keywords:** Periodontitis, Periodontitis

## Abstract

**Design:**

Cross-sectional study

**Case selection:**

Consecutive patient charts (*n* = 220) at Guy’s Dental Hospital between April 2018 and April 2020 were included. The inclusion criteria were adults ≥18 years with a diagnosis of periodontitis (localized or generalized, all stages and grades) and who have received professional mechanical plaque removal (PMPR) by periodontology graduate students. Data of periodontal indices before and after PMPR (6–20 weeks) were also needed to be available. Exclusion criteria included uncontrolled diabetes, pregnancy, medications attributed to drug induced overgrowth, among others.

**Data analysis:**

This retrospective study evaluated the response to periodontal treatment in e-cigarette users and they compared the outcomes to non-smokers, former and current smokers. The primary outcome to evaluate the response to periodontal therapy was ‘need for surgery’. This was defined by the authors as the number of sextants with ≥2 non-adjacent sites with probing depth (PD) ≥ 5 mm after PMPR. Secondary outcomes included periodontal parameters such as number of sextants with ≥1 site with PD ≥ 5 mm, PD, clinical attachment level (CAL), bleeding on probing, recession, and plaque scores.

**Results:**

E-cigarette users and current smokers had similar poorer clinical response to periodontal therapy. Analysis revealed e-cigarette users had more sextants with ‘need for surgery’ as the primary outcome. Pocket closure outcome (PD ≤ 4 mm with no bleeding on probing) were highest in nonsmokers (77.1%), followed by former smokers (74.9%), current smokers (69.4%), and e-cigarette users (66.6%).

**Conclusions:**

E-cigarette users showed less than beneficial response to periodontal therapy compared to non-smokers, who had the best outcome overall.

## A Commentary on

**Shah C, Holtfreter B, Hughes F J, Nibali L**.

Retrospective exploratory study of smoking status and e-cigarette use with response to non-surgical periodontal therapy. *J Periodontol* 2023; **94**: 41–54.

**GRADE Rating**: 

## Commentary

E-cigarettes have gained popularity over the past few years as an alternative to smoking cigarettes and they have been recognized as one of the effective interventions for smoking cessation^1^. Compared to traditional smoking, e-cigarettes have less detrimental effects on health. Nevertheless, e-cigarettes are still considered harmful and could lead to permanent nicotine addiction^2^ and higher risk of myocardial infarction compared to those who do not use e-cigarettes^3^. Smoking is a well-known significant risk factor for poor oral health including periodontitis. Compared to traditional smoking, e-cigarettes have been believed to be less harmful to oral health^4^ including periodontal health^5^. However, there is also evidence that have shown harmful effects of e-cigarettes on oral health compared to those who do not use e-cigarettes^6,7^. With this inconclusive background, it is also not clear how the e-cigarette users respond to periodontal therapy compared to smokers and non-smokers.

In this retrospective study, the authors evaluated the response to PMPR in 220 patients, namely, former smokers (*n* = 60), e-cigarette users (*n* = 20), current smokers (*n* = 20) and non-smokers (*n* = 120). Consecutive records from patients treated in a graduate periodontology clinic were analyzed using recorded pre- and post-PMPR periodontal indices (≥6 weeks). The ‘need for surgery’ was the primary outcome based on the number of sextants with pockets ≥5 mm on ≥2 non-adjacent sites following therapy. Secondary outcome measures of periodontitis including PD, CAL, full mouth bleeding and plaque scores.

Effects of smoking status and treatment comparing the four groups were analyzed and statistically significant differences were found between non-smokers and e-cigarette users and current smokers. E-cigarette users were found to require more surgeries (mean 4.3 surgeries) compared to current smokers (mean 4), former smokers (mean 3.1), and non-smokers (mean 2.4). Among the secondary outcome measures including pocket closure, e-cigarette users had the poorest treatment response compared to other groups.

As rightly pointed out by the authors, the retrospective design of this study along with lack of a standardized protocol and lack of calibration of the clinicians are among the limitations of this study. An unusual endpoint, ‘need for surgery’ is a subjective assessment and does not provide practical clinical relevance.

Practice points

- Educate patients about the potential negative effects of e-cigarette use on periodontal health and treatment outcomes.
- Prospective studies are required to further evaluate response to periodontal therapy in e-cigarette users.
